# The Energy Requirement for Supplemental Greenhouse Lighting Can Be Reduced by Considering ‘Excess’ Light from the Previous Day

**DOI:** 10.3390/plants13050652

**Published:** 2024-02-27

**Authors:** Theekshana C. Jayalath, Marc W. van Iersel, Rhuanito Soranz Ferrarezi

**Affiliations:** Department of Horticulture, University of Georgia, 1111 Miller Plant Sciences Building, Athens, GA 30602, USA; mvanier@uga.edu

**Keywords:** daily light integrals (DLI), plant growth, energy savings, carryover lighting, lettuce

## Abstract

The sunlight greenhouse crops receive varies and is often insufficient for consistent year-round growth in greenhouses. Supplemental lighting is commonly applied in winter, but this practice has a significant energy cost, accounting for 10–30% of operating expenses and impacting greenhouse profitability. Greenhouse lights are traditionally adjusted based on sunlight intensity to meet crops’ daily light requirements. However, if plants can withstand lower daily light integrals (DLI) after a sunny day without reducing the growth, there is potential to reduce the energy required for supplemental lighting and increase the profit. To determine whether excess light received one day can be ‘carried over’ to the next, we grew oakleaf lettuce (*Lactuca sativa* ‘Green Salad Bowl’ and ‘Red Salad Bowl’) under six lighting regimes inside a vertical farm. Plants in all treatments received an average DLI of 15 mol·m^−2^·d^−1^, but DLIs alternated from day-to-day (15/15, 17.5/12.5, 20/10, 22.5/7.5, 25/5, and 27.5/2.5 mol·m^−2^·d^−1^), resulting in DLI fluctuations from 0 to 25 mol·m^−2^·d^−1^. Plants had similar leaf area (~800 cm^2^/plant) and dry weight (~1.8 g/plant) when grown with DLI fluctuations from 0 to 15 mol·m^−2^·d^−1^, while higher DLI fluctuation reduced growth. To confirm this DLI “carrying-over” effect on plants grown under sunlight with supplemental light, we conducted a second study in a greenhouse with ‘Green Salad Bowl’ lettuce. In this study, plants were grown with five different DLI fluctuations (15/15, 16.75/13.25, 18.5/11.5, 20.25/9.75, and 22/8 mol·m^−2^·d^−1^), ranging from 0 to 14 mol·m^−2^·d^−1^, while maintaining an average DLI of 15 mol·m^−2^·d^−1^ in all the treatments. We observed similar leaf area (~750 cm^2^/plant) and dry weight (~1.8 g/plant) in lettuce plants grown with DLI fluctuations from 0 to 10.5 mol·m^−2^·d^−1^. Higher DLI fluctuations reduced growth. Hence, carrying excess light from a sunny to an overcast day is possible within limits. Our study concluded that the DLI requirement can be reduced by approximately 5.25 mol·m^−2^·d^−1^ on the day following a sunny day. By analyzing historical weather data from five US locations, we quantified the potential annual energy savings from incorporating this ‘carrying-over DLI’ concept. This approach resulted in annual energy savings of approximately 75–190 MWh/ha in greenhouse lettuce production. Such reductions in supplemental lighting energy will enhance the profitability and sustainability of the greenhouse industry.

## 1. Introduction

Daily light integral (DLI), the amount of photosynthetically active radiation delivered over a 24-h period, varies daily depending on the weather [1,2]. This inconsistency of sunlight in greenhouses can result in insufficient DLI for crop growth on certain days and excessive DLI on others. Multiple studies strongly recommend maintaining a consistent DLI throughout the growing season to promote optimal crop growth [3,4]. However, the ideal DLI varies depending on the specific crop type [5]. For leafy greens and herbs grown in controlled environments, a recommended DLI of 12 mol·m^−2^·d^−1^ or more is advised, although this can be influenced by factors such as temperature, CO_2_ level, market price, and economic considerations [3]. Therefore, greenhouse growers often provide supplemental light to ensure consistent crop growth and meet the minimum DLI requirement when natural sunlight is insufficient [6,7]. However, the expense of providing supplemental lighting to meet daily constant DLIs can be substantially high. According to a study conducted in Québec City, Canada, the cost of producing 1 kg of lettuce in a greenhouse was approximately $4.66 in US dollars [8]. More than 30% of this production cost can be attributed to the energy cost associated with lighting [9,10,11,12]. This high energy cost can directly impact the profitability and sustainability of the greenhouse operation. Therefore, to enhance the profitability of greenhouse crop production, it is important to reduce this high energy cost associated with supplemental lighting.

Prior studies have developed various control methods for supplemental lighting to maintain a consistent DLI at the canopy level while reducing the associated energy cost [4,6,10]. The study conducted by Albright, Both, and Chiu [4] found that a predictable year-round lettuce production can be achieved by maintaining a consistent DLI throughout the year, using shading and supplemental lighting. Another study incorporated real-time electricity pricing, weather forecasting, and photosynthetic responses to light, demonstrating approximately 25% electricity savings without compromising plant growth [6]. The automated adaptive light emitting diode (LED) lighting control method developed by van Iersel and Gianino [10] to achieve target DLIs indicates 20–92% electricity cost reduction by adjusting the photosynthetic photon flux density (PPFD) threshold considering the instantaneous light received from sunlight. However, these supplemental lighting control mechanisms do not consider the additional DLI plants receive on sunny days, which could reduce the lighting requirements the following day.

In a recent study conducted by Bhuiyan and van Iersel [13], the effect of light fluctuations on the growth of ‘Little Gem’ and ‘Green Saladbowl’ lettuce was evaluated in a growth chamber. They compared 15-min alternating PPFDs with a steady PPFD of 200 µmol·m^−2^·s^−1^. Among the five alternated PPFD treatments, only extreme fluctuations (400/0 and 360/40 µmol·m^−2^·s^−1^) resulted in lower growth and yield than the steady PPFD treatment. However, the treatments with PPFD fluctuations of 320/80, 280/120, 240/160, and 200/200 µmol·m^−2^·s^−1^ exhibited similar leaf area and dry mass. This suggests that lettuce can tolerate a wide range of fluctuating light levels without suppressing its growth. The findings of this study indicate that growers can take advantage of lettuce’s tolerance to fluctuating PPFDs by adjusting the light intensity based on variable electricity prices. This aids growers in reducing the energy costs associated with lighting while maintaining satisfactory crop growth.

Currently, limited research is available regarding identifying long-term (daily) light fluctuations that could further reduce the high energy costs associated with lighting in greenhouse settings. If plants can tolerate fluctuating DLIs, there is a potential to decrease the DLI requirement for greenhouse-grown crops on days following sunny days with excess sunlight. It is assumed that if a crop receives more light than the required DLI on a given day, the DLI requirement for the subsequent day can be reduced without compromising growth as long as the average DLI remains consistent between the two days. Nonetheless, the tolerance of DLI fluctuations between consecutive days may vary depending on the specific crop type. If plants can tolerate fluctuating DLIs, there is a promising possibility of reducing the high energy costs associated with supplemental lighting. Further research in this area would be valuable to explore and maximize the potential benefits. Therefore, this study aims to investigate the tolerance of lettuce (*Lactuca sativa*) to fluctuating DLIs and assess whether such fluctuations result in a change in plant growth.

## 2. Results

The PPFDs measured on a high and a low DLI day of the growth chamber study and the greenhouse study are summarized in Table 1. With increasing the DLI fluctuation from 0 to 25 mol·m^−2^·d^−1^ in our growth chamber study, PPFD ranged from 208 to 383 µmol·m^−2^·s^−1^ on high DLI days and from 208 to 33 µmol·m^−2^·s^−1^ on low DLI days. The treatment with DLI fluctuation of 0 mol·m^−2^·d^−1^ had a constant PPFD of 208 µmol·m^−2^·s^−1^ on both high and low DLI days. The treatment with extreme DLI fluctuations of 25 mol·m^−2^·d^−1^ had alternating PPFDs of 383 µmol·m^−2^·s^−1^ on the high DLI days and 33 µmol·m^−2^·s^−1^ on the low DLI days.

On the other hand, in our greenhouse study, with DLI fluctuations from 0 to 14 mol·m^−2^·d^−1^, the average daily PPFD ranged from 231 to 339 µmol·m^−2^·s^−1^ on high DLI days and from 231 to 123 µmol·m^−2^·s^−1^ on low DLI days (Table 1). The treatment with DLI fluctuation of 0 mol·m^−2^·d^−1^ had an average daily PPFD of 231 ± 12 µmol·m^−2^·s^−1^ (mean ± SD) on both high and low DLI days. The treatment with extreme DLI fluctuations of 14 mol·m^−2^·d^−1^ had alternating PPFDs of 339 ± 12 µmol·m^−2^·s^−1^ on the high DLI days and 123 ± 3 µmol·m^−2^·s^−1^ on the low DLI days. As anticipated, the adaptive lighting control system successfully maintained the designated DLI targets for all treatments throughout the entire duration of the study by adjusting the PPFD accordingly.

### 2.1. Quantum Yield of Photosystem II

**Figure 1:**
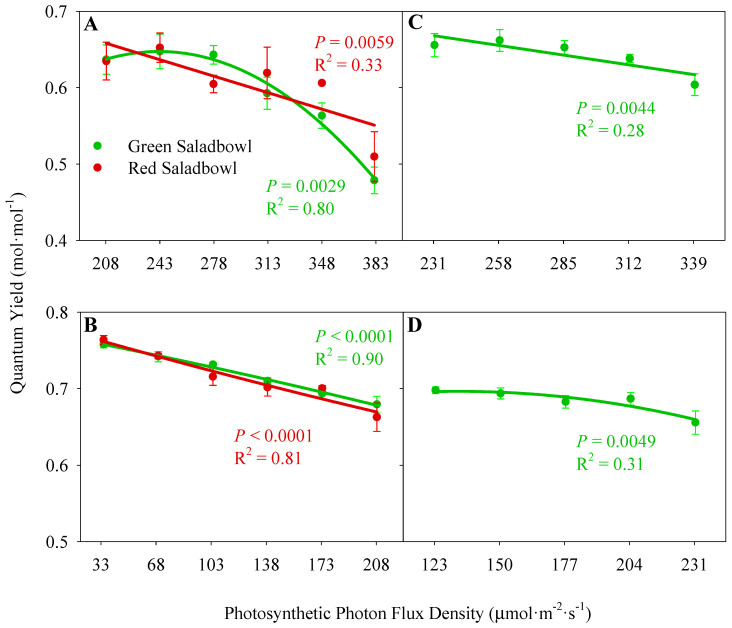
The quantum yield of photosystem II (Φ_PSII_) of lettuce as a function of photosynthetic photon flux density. The Φ_PSII_ was measured on a high DLI day (**A**,**C**) and a low DLI day (**B**,**D**). The Φ_PSII_ of Oakleaf lettuce (*Lactuca sativa*) ‘Green Saladbowl’ and ‘Red Saladbowl’ from the growth chamber study are shown in A and B, while C and D show data from ‘Green Saladbowl’ lettuce grown in the greenhouse study. Each data point represents the average of three plants in the growth chamber study (**A**,**B**) and an average of five plants in the greenhouse study (**C**,**D**). The error bars indicate the standard error of the mean.

In both growth chamber and greenhouse studies, we observed a lower quantum yield of photosystem II (Φ_PSII_) of plants in treatments with higher PPFDs compared to the treatments with lower PPFDs (*p* ≤ 0.0059) (Figure 1). The treatment with the most extreme DLI fluctuation, 25 mol·m^−2^·d^−1^ in the growth chamber study and 14 mol·m^−2^·d^−1^ in the greenhouse study, had the lowest Φ_PSII_ on high DLI days and the highest Φ_PSII_ on low DLI days compared to other treatments (Figure 1).

During the growth chamber study, we noticed a decline in the Φ_PSII_ of ‘Green Saladbowl’ lettuce when PPFD > 278 µmol·m^−2^·s^−1^ on high DLI days (*p* = 0.0059) (Figure 1A). Conversely, ‘Red Saladbowl’ lettuce had a consistent linear reduction in Φ_PSII_ as the PPFD increased from 208 to 383 µmol·m^−2^·s^−1^ (*p* = 0.0029). On low DLI days, both lettuce cultivars indicated a linear reduction of Φ_PSII_ with increasing PPFD (*p* < 0.0001) (Figure 1B). When PPFD increased by 1 mol·m^−2^·s^−1^, the Φ_PSII_ was decreased by 0.003 and 0.005 mol^−1^·mol in ‘Green Saladbowl’ and ‘Red Saladbowl’ lettuce, respectively. During our greenhouse study, with increasing PPFD, we observed a linear reduction of the Φ_PSII_ in ‘Green Saladbowl’ lettuce on the high DLI day (*p* = 0.0044). Φ_PSII_ reduced by 0.005 mol^−1^·mol when PPFD increased by 1 µmol·m^−2^·s^−1^. However, on the low DLI day, we observed that Φ_PSII_ remained stable as PPFD rose from 123 to 204 µmol·m^−2^·s^−1^. When PPFD exceeded 204 µmol·m^−2^·s^−1^, we observed a subsequent decrease in Φ_PSII_ (*p* = 0.0049) (Figure 1C,D).

### 2.2. CO_2_ Assimilation

**Figure 2:**
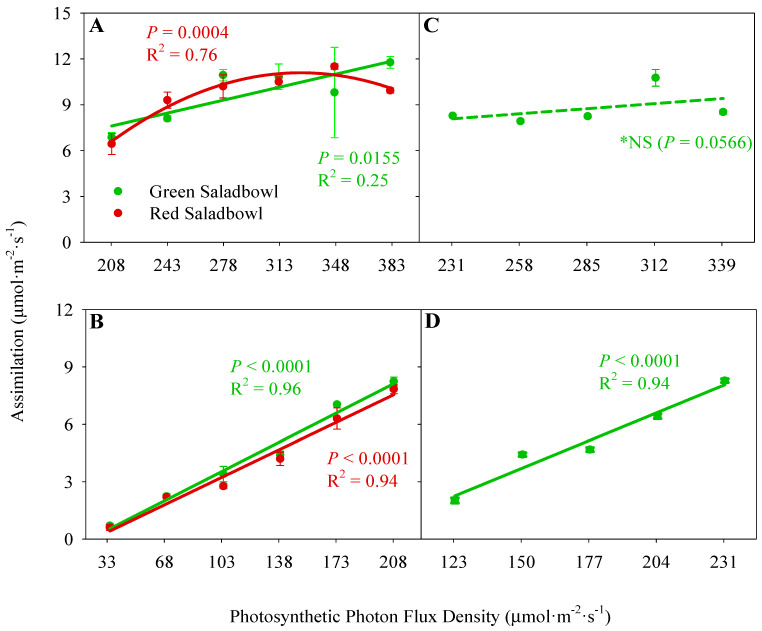
The assimilation of Oakleaf lettuce (*Lactuca sativa*) ‘Green Saladbowl’ and ‘Red Saladbowl’ grown in a growth chamber on a high DLI day (**A**) and a low DLI day (**B**) in response to the photosynthetic photon flux density (PPFD). The assimilation rates of lettuce cultivar ‘Green Saladbowl’ grown in a greenhouse on high (**C**) and low (**D**) DLI days are also presented. Each data point represents the average of three plants in the growth chamber study (**A**,**B**) and five plants in the greenhouse study (**C**,**D**). The error bars indicate the standard error of the mean. *NS = Non-Significant.

In our growth chamber study, we observed an increase in CO_2_ assimilation with an increase in PPFD for ‘Green Saladbowl’ lettuce on both high and low DLI days (*p* = 0.0155 and *p* < 0.0001, respectively) (Figure 2A,B). When PPFD increased by 1 µmol·m^−2^·s^−1^, the CO_2_ assimilation increased by 0.02 and 0.04 µmol·m^−2^·s^−1^ on high and low DLI days, respectively. Likewise, ‘Red Saladbowl’ lettuce also exhibited a linear increase in CO_2_ assimilation as the PPFD increased on the low DLI day (*p* < 0.0001). However, on the high DLI day, CO_2_ assimilation only increased when PPFD rose from 208 to 348 µmol·m^−2^·s^−1^, and further increase reduced the CO_2_ assimilation (*p* = 0.0004) (Figure 2A).

We observed a linear increase in CO_2_ assimilation of greenhouse-grown ‘Green Saladbowl’ lettuce as the PPFD increased on the low DLI day (*p* < 0.0001) (Figure 2D). With increasing of PPFD by 1 µmol·m^−2^·s^−1^, the assimilation increased by 0.05 µmol·m^−2^·s^−1^. The relationship between CO_2_ assimilation and PPFD on the high DLI day was not significant (Figure 2C). However, a noticeable trend indicated that CO_2_ assimilation of greenhouse-grown ‘Green Saladbowl’ lettuce tended to increase by providing more light.

### 2.3. Canopy Growth Rate

**Figure 3:**
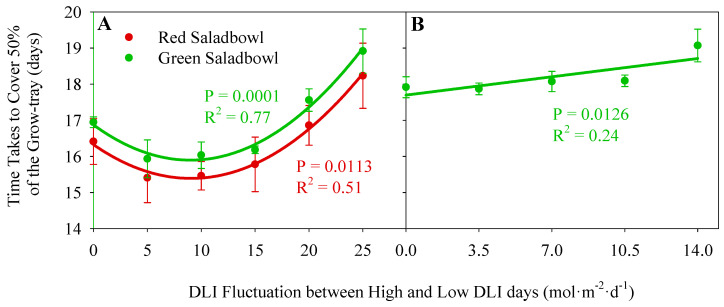
The number of days it took for Oakleaf lettuce (*Lactuca sativa*) ‘Green Saladbowl’ and ‘Red Saladbowl’ canopy to cover 50% of the tray plants were grown during the growth chamber study (**A**) and greenhouse study (**B**), as a function of DLI fluctuation. Each data point represents the average of three replicates in the growth chamber study (**A**) and five replicates in the greenhouse study (**B**). The error bars indicate the standard error.

The canopy growth rate was assessed by calculating the time it took for plants to cover 50% of the tray in which they were grown. This measurement helps determine the speed at which the plants develop their canopy under varying DLI fluctuations. In the growth chamber study, both ‘Green Saladbowl’ and ‘Red Saladbowl’ lettuce cultivars took a similar time to cover 50% of the grow tray by plant canopy when the DLI fluctuation increased from 0 to 15 mol·m^−2^·d^−1^ (*p* = 0.0113) (Figure 3A). Further increase of DLI fluctuation took more time to cover 50% of the tray by plant canopy. This indicates the canopy growth is slower when DLI fluctuation is higher than 15 mol·m^−2^·d^−1^. The ‘Green Saladbowl’ lettuce grown in the greenhouse study indicated a linear increase in the time it took to cover 50% of the tray with increasing DLI fluctuation (*p* = 0.0126) (Figure 3B). Increasing the DLI fluctuation by 1 mol·m^−2^·d^−1^ delayed the time to cover 50% of the tray by 0.1 days.

### 2.4. Light Use Efficiency

**Figure 4:**
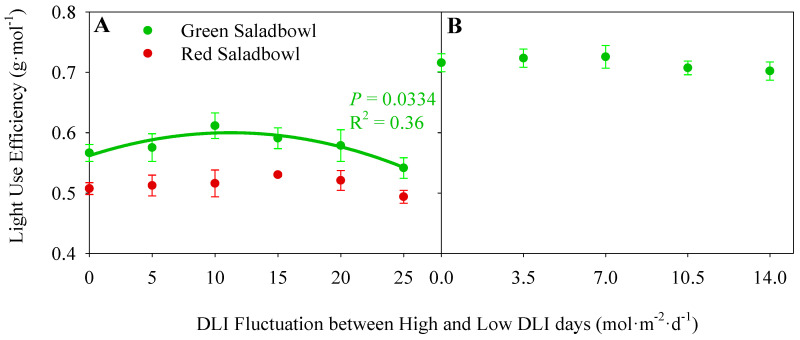
Light use efficiency of Oakleaf lettuce (*Lactuca sativa*), ‘Green Saladbowl’ and ‘Red Saladbowl’ grown in a growth chamber (**A**) and greenhouse (**B**) are presented concerning the level of DLI fluctuation between two days of each treatment. The light use efficiency was calculated by dividing the dry weight of a plant by the total incident light on a plant canopy. Plants grown in the growth chamber and greenhouse study were harvested 28 and 30 days after seeding, respectively. Each data point represents the average of three replicates in the growth chamber study (**A**) and an average of five replicates in the greenhouse study (**B**). The error bars indicate the standard error.

In the growth chamber study, the ‘Green Saladbowl’ lettuce indicated a decrease in LUE when the DLI fluctuation exceeded 20 mol·m^−2^·d^−1^ (*p* = 0.0334) (Figure 4A). However, no significant reduction in LUE was observed for ‘Red Saladbowl’ lettuce grown in the growth chamber study (Figure 4A) or ‘Green Saladbowl’ lettuce grown in the greenhouse study (Figure 4B) when subjected to increasing DLI fluctuation. Nonetheless, both studies exhibited a general trend of reduced LUE at extreme DLI fluctuations when fluctuation exceeded 20 mol·m^−2^·d^−1^ in the growth chamber study and exceeding 10.5 mol·m^−2^·d^−1^ in the greenhouse study.

### 2.5. Leaf Area, Dry Weight, and the Length of the Longest Leaf

**Figure 5:**
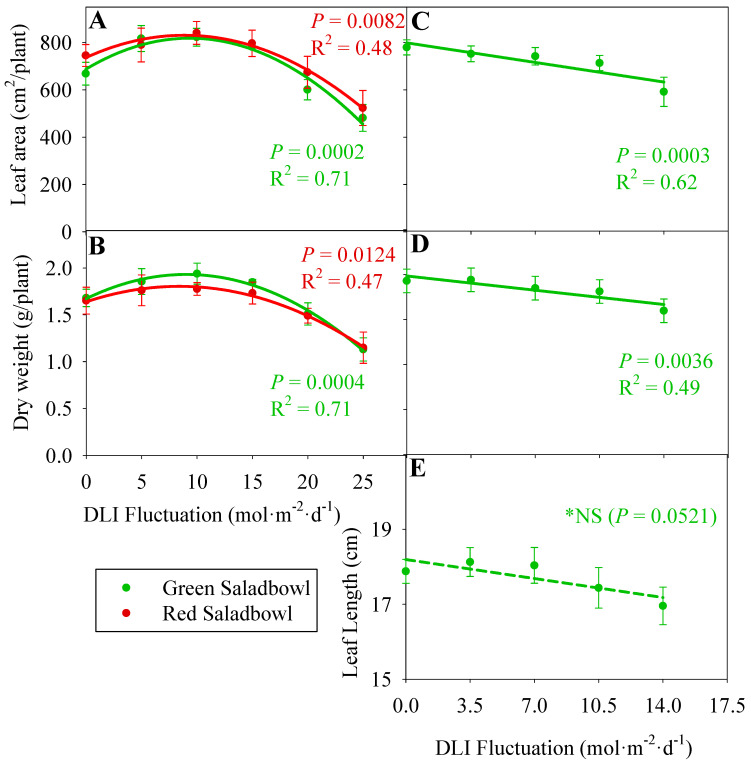
Average leaf area (**A**) and dry weight (**B**) of Oakleaf lettuce (Lactuca sativa) cultivars ‘Green Saladbowl’ and ‘Red Saladbowl’ grown in a growth chamber are presented against the DLI fluctuation of each treatment of the study. Similarly, the average leaf area of a plant (**C**), the dry weight of a plant (**D**), and the average length of the five longest leaves of a treatment (**E**) of ‘Green Saladbowl’ lettuce grown in a greenhouse are presented against the DLI fluctuations. Plants grown in the growth chamber study were harvested at 28 days and in the greenhouse study at 30 days after seeding. Each data point represents the average of three replicates in the growth chamber study (**A**) and an average of five replicates in the greenhouse study (**B**). The error bars indicate the standard error of replicates. *NS = Non-Significant.

In our growth chamber study, we observed that both ‘Green Saladbowl’ and ‘Red Saladbowl’ lettuce cultivars had similar leaf area per plant when DLI fluctuation increased from 0 to 15 mol·m^−2^·d^−1^ (*p* = 0.0002 and 0.0082, respectively) (Figure 5A). However, when the DLI fluctuation exceeded 15 mol·m^−2^·d^−1^, the leaf area per plant decreased for both cultivars. Furthermore, we observed a similar trend in the dry weight data of the lettuce plants in our growth chamber study. When the DLI fluctuation increased from 0 to 15 mol·m^−2^·d^−1^ the dry weight remained relatively similar for both cultivars. However, a further increase in DLI fluctuation reduced the dry weight of both ‘Green Saladbowl’ and ‘Red Saladbowl’ lettuce cultivars (*p* = 0004 and 0.0124, respectively) (Figure 5B). In our greenhouse study, we observed a linear reduction in both leaf area (*p* = 0.0003) and dry weight (*p* = 0.0036) of ‘Green Saladbowl’ lettuce plants as the DLI fluctuation increased from 0 to 14 mol·m^−2^·d^−1^ (Figure 5C,D). For every 1 mol·m^−2^·d^−1^ increase in DLI fluctuation, there was a corresponding decrease of 12 cm^2^ in leaf area and 0.02 g in dry weight per plant.

We observed a trend indicating a decrease in leaf length with increasing DLI fluctuation; however, it was not statistically significant (Figure 5E).

### 2.6. Energy Requirement

**Figure 6:**
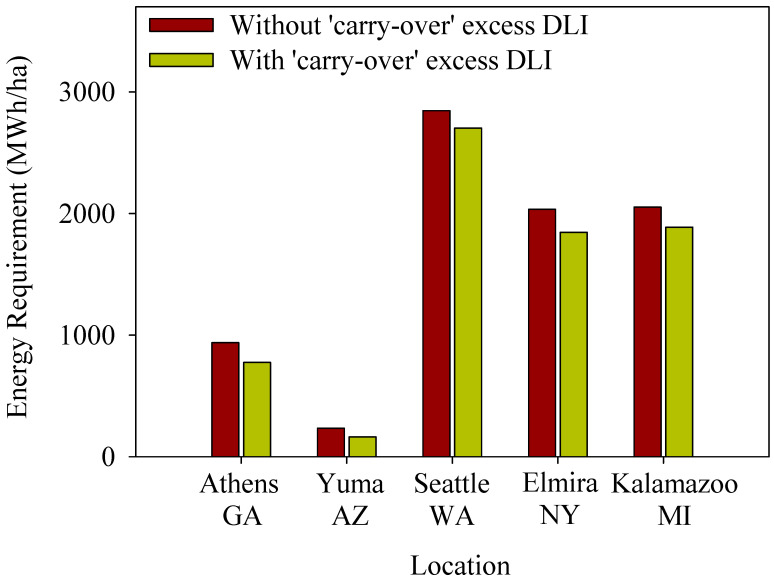
The yearly energy requirement to provide supplemental lighting to reach the daily light integral (DLI) of 17 mol·m^−2^·d^−1^ for an acre of greenhouse lettuce production was calculated for five different locations of the United States using typical meteorological year data (averaged from 2005 to 2015), with and without considering the excess DLI ‘carry-over’ from the previous day. The maximum DLI fluctuation between two consecutive days was kept as 10.5 mol·m^−2^·d^−1^. We considered the efficacy of the lighting fixture as 1.8 mol·m^−2^ and the greenhouse transmission as 70%.

When using the energy calculator to estimate the energy requirement for supplemental LED lighting in greenhouse lettuce production on a hectare scale, we found that significant energy savings can be achieved in all five locations of the United States by implementing our ‘carry-over’ concept of excess DLI from one day to the next (Figure 6). The highest energy savings are projected in Elmira/NY with approximately 189 megawatt hours (MWh) per year for a hectare of greenhouse lettuce production. The locations with the next highest potential energy savings were Kalamazoo/MI (165 MWh/ha), Athens/GA (162 MWh/ha), Seattle/WA (143 MWh/ha), and Yuma/AZ (73 MWh/ha).

## 3. Discussion

The supplemental light in the greenhouse, controlled through the adaptive lighting control system, was able to provide the specified DLIs on both high and low DLI days during the study period. The PPFD was adjusted on each lighting treatment by the datalogger according to the ambient sunlight level to reach the target DLIs (Table 1). This indicates that this approach of providing fluctuation DLIs can be feasible for implementation in a greenhouse environment with fluctuating sunlight.

We observed a consistent decrease in Φ_PSII_ across treatments as light intensity increased on both high and low DLI days (Figure 1). Notably, on high DLI days, the treatments with extreme DLI fluctuations (25 and 14 mol·m^−2^·d^−1^ in the growth chamber and greenhouse study, respectively) had the lowest Φ_PSII_, indicating a reduced fraction of absorbed light being used to drive photosynthesis. This can be associated with the higher PPFDs plants received on high DLI days compared to all the other treatments. At higher PPFDs, an increasing proportion of the PSII reaction centers will close to effectively dissipate absorbed light energy as heat to mitigate potential photodamage to the photosynthetic apparatus [14,15]. Consequently, a reduced fraction of the excitation energy is directed through the PSII reaction centers, resulting in a subsequent reduction in the Φ_PSII_ [16,17]. However, due to the higher flow of electrons in the light reaction of photosynthesis at high light levels, increasing PPFD will enhance the photosynthesis rate up to the light saturation point [14,15]. This will provide more energy for the photosynthesis process and can lead to an increased rate of CO_2_ assimilation. Multiple studies have reported the same: although the Φ_PSII_ decreases with more light provided, the leaf photosynthesis and CO_2_ assimilation increase [15,18,19]. This trend was consistent with our growth chamber study, where we observed a positive correlation between DLI fluctuations and CO_2_ assimilation on high DLI days, attributed to the increased PPFD provided (Figure 2A). However, a slight reduction of CO_2_ assimilation was observed in ‘Red Saladbowl’ lettuce when PPFD exceeded 348 µmol·m^−2^·s^−1^, which may be above the light saturation point of that specific cultivar. While the relationship may not have been statistically significant in our greenhouse study, the trend suggests that higher PPFD levels may still positively impact CO_2_ assimilation in ‘Green Saladbowl’ lettuce under high DLI conditions (Figure 2C). Conversely, on low DLI days, increased DLI fluctuations resulted in reduced assimilation rates due to the limited light availability (Figure 2B,D). Specifically, the treatments with extreme DLI fluctuations exhibited notably low assimilation rates on low DLI days compared to all the other treatments, primarily due to the very low light levels received by the plants (PPFD was 33 ± 0 and 123 ± 7 µmol·m^−2^·s^−1^, on growth chamber and greenhouse studies, respectively). Due to this low light availability at extreme DLI fluctuations, the ‘Green Saladbowl’ and ‘Red Saladbowl’ lettuce had an assimilation rate of 0.7 and 0.6 µmol·m^−2^·s^−1^, in the growth chamber study, respectively. The ‘Green Saladbowl’ lettuce in the greenhouse study had an assimilation rate of 2.0 µmol·m^−2^·s^−1^. Due to these low assimilation rates on low DLI days, the average assimilation between high and low DLI days is much lower at extreme DLI fluctuations compared to the other treatments. This low assimilation may provide limited availability of carbohydrates for the development of new plant tissues and may slower plant growth.

To further assess plant growth with fluctuating DLIs, we used daily PCS to examine the time required for lettuce plants to cover 50% of the tray they were grown in. When the DLI fluctuation exceeds 15 and 10.5 mol·m^−2^·d^−1^ in the growth chamber and greenhouse study, respectively, more time was needed to cover 50% of the tray, indicating a slow canopy growth (Figure 3). Notably, this slow canopy growth was consistently observed on plants subjected to extreme DLI fluctuations in both studies, which aligns with the previously mentioned low averaged CO_2_ assimilation rates observed. A smaller canopy size can potentially reduce the amount of incident light, canopy photosynthesis, and slow growth Klassen et al. [20]. Previous studies have consistently demonstrated this positive correlation between canopy size and plant growth [21,22,23]. A study by Bhuiyan and van Iersel [13] investigated the impact of 15-min light fluctuation on lettuce growth. They observed a lower PCS in the treatment with most extreme PPFD fluctuation, where the light intensity altered between 400 and 0 µmol·m^−2^·s^−1^ at 15-min intervals. They believe this is due to the limited availability of carbohydrates, which are essential for supporting new plant growth. However, all the other PPFD fluctuations had similar PCS, indicating a certain level of tolerance in lettuce towards wide fluctuations in PPFD. Compared with their study, we observed a similar pattern in the PCS of lettuce when DLI fluctuates between two consecutive days. Lettuce could tolerate certain DLI fluctuations, but extreme fluctuations reduced the PCS and canopy growth.

The LUE of lettuce plants remained consistent across different DLI fluctuations in our study (Figure 4). However, there was a notable trend of lower LUE in plants exposed to extreme DLI fluctuations (fluctuation > 20 and 10.5 mol·m^−2^·d^−1^ in the growth chamber and greenhouse study, respectively). This observation can be attributed to the significant decrease in CO_2_ assimilation rates observed on low DLI days (Figure 2) and the limited availability of light to drive photosynthesis on high DLI days due to lower Φ_PSII_ (Figure 1). The combination effect of reduced CO_2_ assimilation and decreased Φ_PSII_ on alternating days may have hindered the efficient utilization of light energy, leading to a lower LUE in lettuce plants under extreme DLI fluctuations. However, the ‘Green Saladbowl’ lettuce had an average LUE of 0.58 g·mol^−1^ in the growth chamber study and 0.71 g·mol^−1^ in the greenhouse study. The lower LUE observed in the growth chamber study may be attributed to the higher fluctuations in DLI experienced by the plants (from 0 to 25 mol·m^−2^·d^−1^) compared to the greenhouse study (from 0 to 14 mol·m^−2^·d^−1^). Previous studies on ‘Green Saladbowl’ lettuce have also reported a range of LUE values from 0.61 to 0.74 g·mol^−1^ [18,24]. In contrast, the average LUE of indoor-grown ‘Red Saladbowl’ lettuce was 0.51 g·mol^−1^, which was comparatively lower than that of ‘Green Saladbowl’. This disparity may be attributed to the absorption of light by anthocyanins, which reduces the availability of light for photosynthesis [25].

When the DLI fluctuation was increased from 0 to 15 mol·m^−2^·d^−1^, the plant’s total leaf area and dry weight in the growth chamber study remained stable (Figure 5A,B). However, once the DLI fluctuation exceeded 15 mol·m^−2^·d^−1^, there was a noticeable decline in the leaf area and the dry weight per plant. This reduction in growth can be attributed to two factors explained earlier. Firstly, we observed a decrease in LUE, and secondly, we noticed a slow increase in the PCS at higher DLI fluctuations. The lower LUE at these higher fluctuations may have contributed to the reduced growth and biomass accumulation in plants. And slower expansion of the canopy size may have decreased the incident light available for photosynthesis, affecting plant growth and productivity. These observations align with the results reported in a previous study investigating growth differences between mizuna (*Brassica rapa* var. *japonica*) and lettuce [18]. They reported that mizuna exhibited faster growth than lettuce due to the larger PCS and higher LUE. The larger canopy size of mizuna allows for increased light capture and utilization, while the higher LUE indicates a more efficient conversion of light energy into biomass. Therefore, the similarities between our study and the previous investigation suggest that PCS and LUE are critical factors that contribute to crop growth differences, not only between mizuna and lettuce but also in response to variations in light conditions, such as increased DLI fluctuations. In the greenhouse study, the ‘Green Saladbowl’ lettuce slightly decreased leaf area and dry weight as the DLI fluctuation increased from 0 to 14 mol·m^−2^·d^−1^ (Figure 5). It is important to note that this decrease in growth is small and may not have substantial implications under moderate DLI fluctuations.

When comparing the overall growth differences observed in both studies, it can be suggested that both lettuce cultivars, ‘Green Saladbowl’ and ‘Red Saladbowl’, can tolerate a DLI fluctuation of 10.5 mol·m^−2^·d^−1^ without experiencing significant suppression of growth. Beyond this point, the growth may be more significantly affected. However, it is important to note that this suggested threshold of 10.5 mol·m^−2^·d^−1^ may vary depending on specific environmental conditions, type of lettuce cultivars, etc.

When we calculate the energy savings that can be achieved by considering a DLI fluctuation of 10.5 mol·m^−2^·d^−1^ between two consecutive days in five US locations, we observed that the highest potential energy savings could be achieved in Elmira/NY, followed by Kalamazoo/MI, Athens/GA, Seattle/WA, and Yuma/AZ (Figure 6). These variations in savings can be attributed to the average DLI received from the natural sunlight in each respective location and the frequency of DLI fluctuations. Yuma/AZ, a region with ample sunlight throughout the year, requires less supplemental lighting for a crop with a DLI of 15 mol·m^−2^·d^−1^. Consequently, the potential for energy savings on lettuce production in Yuma, AZ, is comparatively lower than in the other locations. On the other hand, places like Elmira, NY, which receives lower levels of natural sunlight year-round, would require more supplemental lighting. The energy requirement for supplemental lighting to sustain year-round greenhouse lettuce production in Elmira, NY, is ten times higher than in Yuma, AZ. As a result, implementing the concept of DLI fluctuation in Elmira, NY, would yield significant energy savings for lettuce production due to the higher reliance on supplemental lighting.

Our findings indicate that maintaining a consistent DLI throughout the growing season may not be necessary for lettuce, as proposed by Albright, Both, and Chiu [4]. As suggested in their study, the use of movable shades to ensure a constant DLI throughout the season may be optional. Instead, our results suggest that allowing lettuce crops to receive higher DLIs on sunny days can compensate for lower DLIs on subsequent days up to a certain threshold. Significant growth reduction is unlikely to occur as long as the average DLI between two consecutive days aligns with the DLI requirement of a specific crop. Notably, a study by Mayorga-Gomez and van Iersel [26] focused on investigating the impact of the DLI ‘carryover’ concept over several days following a day with high DLI exposure. The study found that this DLI ‘carryover’ effect could be extended for up to five days without causing significant growth reduction. Consequently, incorporating this approach has the potential to significantly lower the energy cost related to supplemental lighting for greenhouse operators. When formulating algorithms for the energy-efficient control of LED lighting systems in greenhouse supplementary lights, it is imperative to factor in this concept.

## 4. Materials and Methods

We conducted two separate studies to investigate the impact of DLI fluctuations on plant growth, The first study was to assess the potential ‘carry-over’ effect of excessive light received on one day to the subsequent day. This study was conducted in a growth chamber environment with sole-source lighting. Subsequently, to validate the observed DLI ‘carry-over’ effect on the first study, we grew plants under natural sunlight with supplemental lighting in a greenhouse setting. This second study aimed to confirm whether the DLI fluctuation tolerance observed in lettuce plants is also exhibited under natural sunlight.

### 4.1. Experimental Setup

#### 4.1.1. Alternating DLI in Growth Chamber

This study was conducted in a 4.4 m wide and 4.1 m long growth chamber to identify plant growth responses due to fluctuating DLI. The growth chamber had three 2.4 m long × 0.6 m wide × 2.2 m high metal shelving racks. Each rack had three shelves with a 0.6 m × 2.4 m ebb-and-flow tray on each shelf. Each ebb-and-flow tray was divided into two 1.2 m long sections using Styrofoam sheets wrapped in reflective aluminum film. Each rack had six individual 1.2 m long × 0.6 m wide × 0.6 m high growing sections. Two 1.1 m-long white LED light fixtures (RAY series with Physiospec indoor spectrum; Fluence Bioengineering, Austin, TX, USA), mounted 0.4 m above the bottom of the ebb-and-flow tray in each section (Figure S1).

The PPFD of those six sections was controlled using a datalogger (CR6; Campbell Scientific, Logan, UT, USA) connected to dimmable drivers that powered the LED fixtures. The LED fixtures were on for 16 h·d^−1^. The chamber temperature was maintained using a top-mount refrigeration system, and a dehumidifier was placed inside the chamber to reduce the humidity. Temperature and relative humidity measurements were collected every ten seconds with a probe (HMP50; Vaisala, Helsinki, Finland) connected to the datalogger, and vapor pressure deficit (VPD) was calculated. The average temperature and the VPD inside the growth chamber during the study were 24.2 ± 0.2 °C and 1.1 ± 0.2 kPa (mean ± SD), respectively. The CO_2_ level inside the growth chamber was measured and maintained at 800 μmol·mol^−1^ by triggering a solenoid valve to open and release CO_2_ from a compressed gas cylinder for 1-s intervals whenever the CO_2_ concentration dropped below 800 μmol·mol^−1^, using a CO_2_ transmitter (GMC20; Vaisala, Helsinki, Finland) connected to the datalogger. The average CO_2_ level inside the growth chamber during the study was 816 ± 28 μmol·mol^−1^ (mean ± SD).

We placed two groups of 15 10-cm^2^ pots in each of the 18-growing sections, with a plant density of 42 per m^2^. Those pots were filled with a soilless substrate [80% peat: 20% perlite (*v*/*v*) (Fafard 1P; SunGro Horticulture, Agawam, MA, USA)]. Oakleaf lettuce ‘Green Salad Bowl’ was seeded in half the pots, and ‘Red Salad Bowl’ in the other half. To prevent algae growth on the surface of the substrate, the top 1 cm of each pot was filled with calcined clay (Turface^®^ Pro League Elite, Profile Products LLC, Buffalo Grove, IL, USA). Plants were grown under six treatments with different DLI fluctuations within two consecutive days (Figure S2A). Each treatment had a high DLI day followed by a low DLI day. High and low DLI days were alternated during the experiment as 15/15, 17.5/12.5, 20/10, 22.5/7.5, 25/5, and 27.5/2.5 mol·m^−2^·d^−1^. Therefore, DLI fluctuations between the two days of each treatment were 0, 5, 10, 15, 20, and 25 mol·m^−2^·d^−1^, respectively. However, all treatments received an average DLI of 15 mol·m^−2^·d^−1^ between the two days. Plants were sub-irrigated daily for 5 min with a nutrient solution containing 100 mg·L^−1^ N made with a 15N–2.2P–12.45K water-soluble fertilizer (Peters Excel 15–5–15 Cal-Mag Special; Everris NA Inc., Dublin, OH, USA). Fertilizer solution final nutrient content in mg/L: N = 150, NO_3_ = 118, NH_4_ = 11, Urea = 21, P = 21.85, K = 124.50, Ca = 50, Mg = 20, B = 0.187, Cu = 0.187, Fe = 0.750, Mn = 0.375, Mo = 0.075, and Zn = 0.375.

#### 4.1.2. Alternating DLI in the Greenhouse

To further investigate the growth responses due to fluctuating DLIs with supplemental light, we conducted a follow-up study on a 9 m long × 1.5 m wide bench located in a glass-covered greenhouse in Athens, GA, USA (lat. 33°57′26.676″ N, long. 83°22′36.48″ W). The greenhouse bench was divided into five blocks using five 1.5 m long × 0.9 m wide ebb-and-flow trays. Each tray was further separated into five sections using vertical aluminum panels to avoid light pollution among the treatments. To provide supplemental light to reach the daily target DLI of each treatment, 1.1 m-long white LED light bars (RAY series with Physiospec Greenhouse spectrum; Fluence Bioengineering) were hung 38 cm above the ebb-and-flow tray. The center block had five quantum sensors (SQ-500-SS; Apogee Instruments, Logan, UT, USA) placed 0.15 m above the ebb-and-flow tray, one in each of the five lighting treatments. The distance between the LED light bars and quantum sensors was 0.23 m. Those five quantum sensors were connected to a datalogger (CR1000, Campbell Scientific, Logan, UT, USA) to measure and record the instantaneous PPFD in each section. The data logger was connected to a 4-channel analog output module (SDM-AO4A, Campbell Scientific, Logan, UT, USA) to precisely control the dimming signal sent out to four of the LED drivers. The dimming signal sent out from each channel of the analog output module was independently adjusted from 0 to 10 V direct current (DC). Each channel was connected to a separate LED driver, and each driver powered five LED light bars, one in each block. Those five LED bars powered by a single driver were used as five replicates of a treatment. The fifth driver was controlled by a dimming signal sent by the datalogger through a pulse width modulation (PWM) control board (XY-C-1215; PanlongIC, Weifang, China). We used an adaptive lighting control system to precisely control the DLI plants receive each day (Weaver and van Iersel, 2020). High and low DLI days of each treatment were alternated as 15/15, 16.75/13.25, 18.5/11.5, 20.25/9.75, and 22/8 mol·m^−2^·d^−1^ with DLI fluctuations of 0, 3.5, 7, 10.5, and 14 mol·m^−2^·d^−1^ respectively. Similarly to our indoor study, the average DLI between two days was maintained at 15 mol·m^−2^·d^−1^ throughout the study. Supplemental light was provided for 18 h per day. A 60% shade net was placed above the supplemental lights to prevent exceeding the DLI target from sunlight (Figure S2B).

We grew ten plants of oakleaf lettuce, ‘Green Salad Bowl’, in each section using 10-cm square pots, with a plan density of 38 per m^2^. Those pots were filled with a soilless substrate [80% peat: 20% perlite (*v*/*v*) (Fafard 1P; SunGro Horticulture)]. Similarly to the indoor study, the top 1 cm of each pot was filled with calcined clay (Turface^®^ Pro League Elite) to prevent algae growth on the surface of the substrate. Each treatment was repeated five times within the five ebb-and-flow trays. Plants were subirrigated daily for 5 min with the same nutrient solution containing 100 mg·L^−1^ N made with a 15N–2.2P–12.45K water-soluble fertilizer (Peters Excel 15–5–15 Cal-Mag Special; Everris NA). The average temperature and VPD inside the greenhouse were 24.2 ± 0.2 °C and 1.1 ± 0.2 kPa (mean ± SD), respectively.

### 4.2. Data Collection and Calculations

In both studies, we collected canopy images of plants twice a week throughout the growing period, using a chlorophyll fluorescence imaging setup. Images were collected on a group of plants, 15 in our growth chamber study and 10 in our greenhouse study. For the fluorescence imaging, we used a monochrome camera (CM3-U3-31S4M-CS, Chameleon3 USB3 camera, FLIR Systems, Arlington, VA, USA) with a 665 nm longpass filter (LP665 Dark Red Longpass Filter; Midopt Midwest Optical Systems, Palatine, IL, USA) attached to the lens. The camera was mounted facing downward inside a 1.2 m × 0.6 m × 1.5 m grow tent. A blue LED panel was mounted inside the tent next to the camera to excite chlorophyll and induce fluorescence. The camera captured reemitted light from chlorophyll fluorescence. Those images were then analyzed with an image analyzing software to determine the projected canopy size (PCS). Those values were plotted against time, and sigmoidal curves [f = a/(1 + e^(x×x0)/b^)] were fitted (SigmaPlot 11.0, Systat Software, San Jose, CA, USA). Using the coefficients of the sigmoidal equation, the daily PCS was estimated (Microsoft Excel 365, Microsoft Corporation, Redmond, WA, USA). This was done for all individual treatments and replicates (R^2^ > 0.99). These estimated PCSs determined the time it took to cover 50% of the growing tray area by plant canopy.

In addition, the daily PCS data were multiplied by the DLI received in each corresponding treatment, and the daily incident light per plant was calculated. The total incident light on a plant canopy throughout the growing period was calculated by adding those daily incident light values. By dividing the dry weight of a plant by the total incident light on a plant canopy, the light use efficiency (LUE) was calculated. In both studies, a day before the harvest, the leaf chlorophyll content index (CCI) of plants was measured using a chlorophyll meter (CCM-200 plus; Apogee Instruments, Logan, UT, USA) on uppermost fully expanded leaves.

In addition, the quantum yield of photosystem II (Φ_PSII_) and CO_2_ assimilation of a randomly selected plant in each treatment were also measured using a leaf gas exchange system equipped with a chlorophyll fluorometer (CIRAS-3 Portable Photosynthesis System: PP Systems, Amesbury, MA, USA). The white LED light in the leaf cuvette was used to provide the corresponding PPFD (Table 1) of each treatment during the measurements. Both Φ_PSII_ and CO_2_ assimilation measurements were taken on the last low DLI day and the last high DLI day of the experiments.

Both ‘Green Salad Bowl’ and ‘Red Salad Bowl’ lettuce grown in our indoor study were harvested 28 days after seeding, and ‘Green Salad Bowl’ lettuce grown in the greenhouse study was harvested 30 days after seeding. During the harvest, the total leaf area of plants in each treatment was measured using a leaf area meter (LI-3100 leaf area meter; LI-COR Biosciences, Lincoln, NE, USA), and the dry weight of those plants was measured after drying them at 80 °C for 7 days. The average leaf area and the dry weight per plant were calculated by dividing the total measured values by the number of plants in each treatment. In addition to these data, for our greenhouse study, we measured the length of the longest leaf of five randomly selected lettuce plants in each treatment.

At the end of the study, we used typical meteorological year data (averaged from 2005 to 2015) to calculate how much light energy can be saved by adapting the DLI carryover concept for greenhouse lettuce production. We conducted an analysis to determine the energy requirements for an acre of greenhouse lettuce production in five different locations in the United States (Athens/GA, Yuma/AZ, Seattle/WA, Elmira/NY, and Kalamazoo/MI). The locations were chosen specifically for their diverse and contrasting light conditions. The average DLIs of these five locations during the summer and winter months are mentioned in Table 2. The analysis involved two scenarios: one considering the concept of DLI carryover and the other without considering it (Microsoft Excel 365, Microsoft Corporation, Redmond, WA, USA). For the calculation, we assumed lettuce plants need an average DLI of 17 mol·m^−2^·d^−1^ throughout a 30-day growing period. When the DLI of sunlight exceeds 17 mol·m^−2^·d^−1^, the DLI target for the subsequent day is set to lower than 17 mol·m^−2^·d^−1^. The average, typical meteorological year data was used to identify the overcast days followed by sunny days with DLI of more than 17 mol·m^−2^·d^−1^. The calculation is based on the additional light plant received on the previous sunny day. The maximum DLI fluctuations between two consecutive days were set as 10.5 mol·m^−2^·d^−1^. We used the efficacy of the lighting fixture as 1.8 µmol·J^−1^ and the greenhouse sunlight transmission as 70% for the calculation.

### 4.3. Experimental Design and Statistical Analysis

The experimental design of the growth chamber studies was a randomized complete block design with six treatments and 3 blocks. Each metal shelving rack was a block. The experiment unit was a group of 15 plants. Similarly, the experimental design of our greenhouse study was also a randomized complete block design, but with five treatments and five blocks. The experiment unit of the greenhouse study was a group of ten plants.

Both quantum yield and assimilation data were analyzed by regression, considering the average PPFD of high and low DLI days as the independent variables (α < 0.05), using statistical software (SAS University Edition; SAS Institute, Cary, NC, USA). To test the effects of DLI fluctuations on CCI, LUE, PCS, leaf area, and dry weight of plants, regression analyses were conducted with DLI fluctuation as the independent variable (α < 0.05).

## 5. Conclusions

The tolerance of lettuce to DLI fluctuations of 10.5 mol·m^−2^·d^−1^ observed in both indoor and greenhouse settings presents a significant opportunity for greenhouse lettuce growers to potentially save a substantial amount of money on energy costs associated with supplemental lighting. By leveraging this concept, growers can potentially reduce the need for constant and precise light intensity control, leading to energy savings and cost reductions. The specific amount of energy savings may vary depending on the location and local climatic conditions, but overall, adopting this concept can contribute significantly to reducing energy consumption in greenhouse lettuce production across different regions in the United States. However, it is essential to emphasize that before implementing this concept, it is crucial to identify the optimal DLI fluctuation levels for specific crops and cultivars. Each crop and cultivar may have different light requirements and responses, and it is important to ensure that they receive sufficient light for their growth and development.

## Figures and Tables

**Table 1:** The corresponding PPFDs on high DLI days and low DLI days of each treatment in the growth chamber and greenhouse studies are mentioned. The PPFD values indicate the average PPFD over the entire day. The PPFD did not vary in the growth chamber study but was not constant throughout the day in the greenhouse study. The standard deviation from the average PPFDs of the greenhouse study is also mentioned.

		DLI Fluctuation (mol·m^−2^·d^−1^)					
Growth chamber		0	5	10	15	20	25
	High DLI-day PPFD (µmol·m^−2^·s^−1^)	208	243	278	313	348	383
	Low DLI-day PPFD (µmol·m^−2^·s^−1^)	208	173	138	103	68	33
		DLI Fluctuation (mol·m^−2^·d^−1^)					
Greenhouse		0	3.5	7	10.5	14	
	High DLI-day PPFD (µmol·m^−2^·s^−1^)	231 ± 12	258 ± 4	285 ± 4	312 ± 0	339 ± 0	
	Low DLI-day PPFD (µmol·m^−2^·s^−1^)	231 ± 3	204 ± 1	177 ± 5	150 ± 8	123 ± 7	

**Table 2:** The average daily light integrals (DLI) during the summer (June–August) and winter (December–February) months of the five US locations selected for the energy savings calculation.

Location	Summer DLI Average (mol·m^−2^·d^−1^)	Winter DLI Average (mol·m^−2^·d^−1^)
Athens/GA	45.7	20.0
Yuma/AZ	52.3	24.7
Seattle/WA	44.1	8.8
Elmira/NY	34.9	14.3
Kalamazoo/MI	39.6	11.6

## Data Availability

All data are available upon reasonable request.

## Supplementary Materials

The following supporting information can be downloaded at: https://www.mdpi.com/article/10.3390/plants13050652/s1, Figure S1: Diagram of the experimental setup in one of the metal shelving racks of the growth chamber.; Figure S2: (A) Overview of the six lighting treatments within one metal shelving rack of the growth chamber study. (B) Overview of the five lighting treatments in the greenhouse study.
