# Supplementary material for: The Energy Requirement for Supplemental Greenhouse Lighting Can Be Reduced by Considering ‘Excess’ Light from the Previous Day
Source: Plants (Basel). 2024 Feb 27;13(5):652. doi: 10.3390/plants13050652 (PMC10934181; doi:10.3390/plants13050652)

Figure S1. Diagram of the experimental setup in one of the metal shelving racks of the growth chamber. Each shelving rack has six sections used for the six lighting treatments of the study. Each section has two light emitting diode (LED) lighting fixtures and 15 plants of Oakleaf lettuce (*Lactuca sativa*) ‘Green Saladbowl’ and 15 plants of ‘Red Saladbowl’. This setup was repeated in three shelving racks within the same growth chamber.

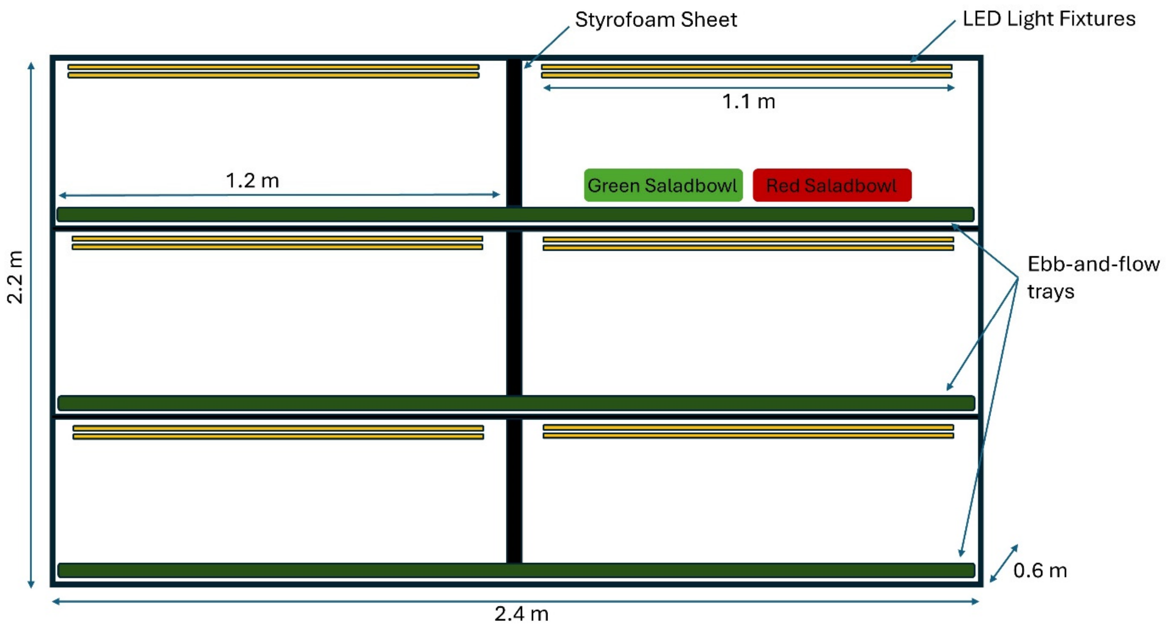

Figure S2. (A) Overview of the six lighting treatments within one metal shelving rack of the growth chamber study. Each section had 15 plants of Oakleaf lettuce (*Lactuca sativa*) ‘Green Saladbowl’ and 15 plants of ‘Red Saladbowl’. This setup was repeated in three shelving racks within the same growth chamber. (B) Overview of the five lighting treatments in the greenhouse study. Each section had 10 plants of ‘Green Saladbowl’ lettuce. These treatments were repeated five times within the same greenhouse space.

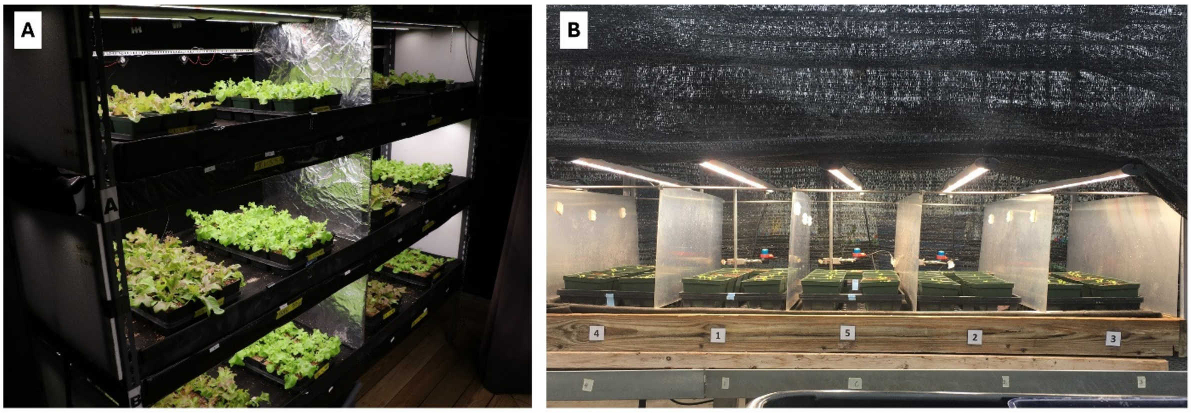

Supplement: Supplementary file 1 [file plants-13-00652-s001.zip › plants-2870570-supplementary.pdf]
